# TP53 mutation‐related senescence is an indicator of hepatocellular carcinoma patient outcomes from multiomics profiles

**DOI:** 10.1002/SMMD.20230005

**Published:** 2023-04-13

**Authors:** Yu‐Yan Chen, Zheng‐Yi Zhu, Tao Ma, Lu Zhang, Jing Chen, Jia‐Wei Jiang, Cui‐Hua Lu, Yi‐Tao Ding, Wen‐Xian Guan, Nan Yi, Hao‐Zhen Ren

**Affiliations:** ^1^ Department of Hepatobiliary Surgery Affiliated Drum Tower Hospital of Nanjing University Medical School Nanjing China; ^2^ Department of Gastroenterology Affiliated Hospital of Nantong University Nantong China; ^3^ Nanjing Drum Tower Hospital Clinical College of Jiangsu University Nanjing China; ^4^ Department of General Surgery Affiliated Drum Tower Hospital of Nanjing University Medical School Nanjing China

**Keywords:** hepatocellular carcinoma, immunotherapy, senescence, senescence‐associated secretory phenotype, TP53 mutation

## Abstract

TP53 mutation frequently occurs in hepatocellular carcinoma (HCC). Senescence also plays a vital role in the ongoing process of HCC. P53 is believed to regulate the advancement of senescence in HCC. However, the exact mechanism of TP53 mutation‐related senescence remains unclear. In this study, we found the TP53 mutation was positively correlated with senescence in HCC, and the differential expressed genes were primarily located in macrophages. Our results proved that the risk score could have an independent and vital role in predicting the prognosis of HCC patients. In addition, HCC patients with a high risk score may most probably benefit from immune checkpoint block therapy. We also found the risk score is elevated in chemotherapy‐treated HCC samples, with a high level of senescence‐associated secretory phenotype. Finally, we validated the risk‐score genes in the protein level and noticed the risk score is positively related with M2 polarization. Of note, we considered that the risk score under the TP53 mutation and senescence is a promising biomarker with the potential to aid in predicting prognosis, defining tumor environment characteristics, and assessing the benefits of immunotherapy for HCC patients.

## Graphical abstract

The TP53 mutation‐related senescence is a promising biomarker with the potential to aid in predicting prognosis, defining tumor environment characteristics, and assessing the benefits of immunotherapy for HCC patients.

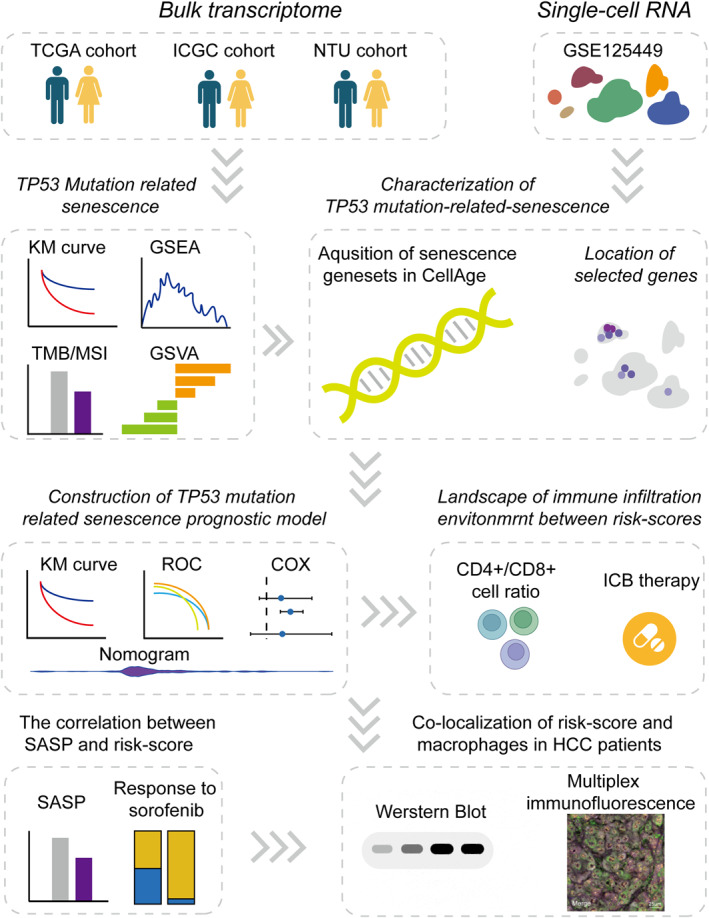

1

**Key points**

- Senescence is a character of TP53 mutation in hepatocellular carcinoma (HCC) patients.
- The SASP generated by chemotherapy caused poor prognosis of HCC patients.
- The TP53 mutation‐related senescence accelerates the M2 polarization of macrophages in HCC patients.

## 2 INTRODUCTION

Hepatocellular carcinoma (HCC) remains the most common type among multiple hepatic cancers and the fifth primary cause of cancer‐related mortality worldwide, especially having the most rapidly growing mortality rate in Asia.^1^ Currently, liver resection with adjuvant chemotherapy has become a routine treatment.^2^ Sorafenib, which is the earliest approved first‐line treatment for advanced liver cancer, has a certain effect on the treatment of HCC. However, according to clinical observations, the overall treatment efficiency for HCC is only 5%.^3^ This is due to the considerable heterogeneity of clinical treatment of HCC, which is validated in genomics and epigenetics.^4^ Hence, underscoring the need for novel predictive markers of HCC subtypes can help us explore the potential for therapeutic targeting. With the development of high‐throughput sequencing, molecular classifications of HCC in numerous studies provide the basis for the development of clinically targeted drugs. For instance, Coulouarn et al. found that HCC defined by the advanced TGF‐β signature is less differentiated and more sensitive to oxidative damage.^5^ Chiang et al. identified five types by hierarchical clustering, containing CTNNB1, proliferation, IFN‐related, novel type defined by polysomy on Chromosome 7 and unannotated type, while some types can better respond to antitumor angiogenesis therapy.^6^ Nevertheless, these defined classifications face significant barriers in the clinical translation and limitations in relevant HCC patients.

TP53 is frequently mutated in HCC, and most of these alterations are missense mutations that result in the upregulation of Mutant p53.^7^ The wild‐type TP53 protein can inhibit the division of cells with DNA damage and chromosomal aberrations, thereby preventing the transmission of the aberrations to daughter cells and has a broad spectrum tumor suppressor effect. On the contrary, the TP53 mutation can result in centrosome amplification processes, cellular aneuploidy proliferation processes, and chromosomal instability.^8^ Previous studies also found that TP53‐mutated HCC cells can evade immune surveillance to escape the fate of apoptosis.^9^ These findings suggest that TP53 mutation is closely associated with the oncogenesis and development of HCC. Recently, high TP53 mutation frequency was shown to not only be associated with the poor prognosis of HCC patients but also to be positively associated with vascular invasion, clinical stage and other clinical characteristics.^10^, ^11^, ^12^ Understanding the underlying mechanism of TP53 mutation in the oncogenesis and development of HCC is of great help to our clinical treatment. However, there are very few studies on the pathogenesis of TP53 mutation in HCC.

Senescence, which was studied for the first time in the *Caenorhabditis elegans* system nearly 40 years ago, has been listed as one of the hallmarks of cancer so far.^13^, ^14^ Tumor cell senescence is an irreversible form of cell cycle inhibition, which may evolve as a protective mechanism to maintain tissue homeostasis.^15^ In general, cellular senescence is an important tumor suppression method for HCC. However, some studies have also shown that senescent cells can excrete multiple cytokines, chemokines, and matrix remodeling enzymes, which could shape the tumor environment through the senescence‐associated secretory phenotype (SASP).^16^ These secreted factors not only have the potential to induce immune cells to clear senescent cells but also have the potential to promote tumor progression.^17^ Due to the diverse and promiscuous function, much attention has been drawn to senescence. Wang et al. found that XL413, a cell division cycle kinase inhibitor, can induce senescence in TP53‐mutated HCC cells, and then Sertraline was specifically used to kill this type of senescent cells, thereby improving HCC patients' survival.^18^ Although this study provides us with the possibility of treating liver cancer through cellular senescence, the path to clinical translation remains unclear due to its lack of clear targets.

Here, we showed that the TP53‐mutated samples were remarkably enriched in the senescence processes and then sought to develop a TP53 mutation‐related senescence prognostic risk‐score model for HCC on the basis of the The Cancer Genome Atlas (TCGA) cohort. Then the model stability and applicability were validated in the International Cancer Genome Consortium (ICGC) cohort. Subsequently, we further verified the risk score is strongly correlated with various tumor signaling pathways. In addition, we observed that the risk score is positively concerned with SASP generated in chemotherapy. Finally, it is validated that high risk score is capable of predicting not only the outcomes of HCC patients but also the sensitivity to immune checkpoint block (ICB) therapy.

## 3 METHODS

### 3.1 Public data collection and processing

In this study, several HCC cohorts were included for analysis. The TCGA‐LIHC cohort, which included 302 samples with transcriptome data, detailed clinical information, copy number variation (CNV) data, and mutation data, was used as an explorative dataset. R package “TCGAbiolinks,” “ChAMPdata,” and “maftools” were used to download and process these data.^19^ The ICGC‐LIRI‐JP cohort, which included 240 samples, with transcriptome data, detailed clinical information, methylation data, and mutation data, was used as a validation dataset (https://dcc.icgc.org/). Table 1 exhibited the specific clinical information of TCGA and ICGC cohorts. In addition, GSE109211, which included 67 samples treated with sorafenib and 73 samples treated with placebo was downloaded to detect the drug response of sorafenib. Finally, single‐cell transcriptome data from GSE125449, which included nine HCC samples, were downloaded to find where these selected genes express (https://www.ncbi.nlm.nih.gov/geo/).

**TABLE 1:** Clinical characteristics of HCC patients in TCGA, ICGC and NTU databases between the low and high risk‐score groups.

Character	TCGA			ICGC			NTU		
	Low	High	*p* Value	Low	High	*p* Value	Low	High	*p* Value
TP53			＜0.001			＜0.001			
TP53 wild	125	76		96	56				
TP53 mutation	26	75		24	64				
Age (years)			0.019			0.07			0.009
≤65	79	100		44	41		27	38	
＞65	72	51		76	79		15	4	
Gender			0.169			0.374			0.03
Female	40	52		27	34		13	4	
Male	111	99		93	86		29	38	
*T* stage			0.045						
T1	83	65							
T2	39	42							
T3	21	39							
T4	7	5							
*N* stage			0.604						
N0	96	108							
N1	2	1							
*M* stage			0.236						
M0	106	114							
M1	2	0							
TNM stage			0.022			0.944			0.02
I	78	63		19	17		18	8	
II	36	38		55	54		11	11	
III	24	43		35	39		6	4	
IV	2	0		11	10		7	19	
Histological grade			0.01						
G1	22	16							
G2	85	66							
G3	37	61							
G4	3	8							
BCLC stage									0.190
A							14	7	
B							12	13	
C							16	22	

Abbreviations: HCC, hepatocellular carcinoma; ICGC, International Cancer Genome Consortium; NTU, Nantong University; TCGA, The Cancer Genome Atlas.

For the TCGA dataset, original count data was utilized to distinguish the differentially expressed genes between the TP53 mutation group and the TP53 wild group. The normalized sequencing data (FPKM) was used to evaluate the values of each group. Transcripts per kilobase million (TPM) values were transformed from FPKM and used for further estimate and the CIBERSORT algorithm. For the ICGC dataset, which was based on the Illumina HiSeq platform, the same as TCGA, the FPKM data was used to evaluate the values of each group. For GSE109211, microarray data was adjusted by R package “Limma” to remove the batch effect.^20^ The detailed methods for GSE125449 are described in the part “Single cell data analysis” individually.

### 3.2 Differentially expressed genes and functional enrichment

According to the screening criteria for differentially expressed genes (DEGs): |log2Foldchange| >1, the false discovery rate <0.05, the DEGs between the TP53 mutation group and TP53 wild group in the TCGA HCC dataset were screened via the Deseq2 package.^21^ The Gene Ontology (GO) function was employed to assess the DEGs by “clusterProfiler” R package. The corrected adjusted *p* value was set to <0.05 as the screening criterion.

### 3.3 Gene set enrichment analysis and gene set variation analysis

For the sake of a better understanding of the enriched signaling pathways between the TP53 mutation group and TP53 wild group in the TCGA HCC dataset, gene set enrichment analysis (GSEA) was applied to investigate the signaling pathways enriched in the TP53 mutation and TP53 wild subgroups using the R package, including “clusterProfiler” and “enrichplot.”^22^ The KEGG database was selected as a reference gene set. Gene set variation analysis (GSVA) is an analysis of gene set variation using microarrays and RNA sequencing.^23^ Various enriched pathways from hallmarks, C2 and C5 reference gene sets, contained with senescence and tumor microenvironment‐related pathways, were calculated and transformed into the expression of each sample individually.

### 3.4 Prognostic model establishment and validation

Using the Kaplan–Meier survival package in R, we utilized the univariate Cox method to examine the relationship between the 15 senescence‐related DEGs and survival status in the TCGA HCC cohort to filter genes with prognostic significance. With the R package “glmnet,” we then estimated the risk score for five genes based on LASSO Cox regression. The gene signatures were filtered using the optimum punishment parameter (λ) determined using tenfold cross‐validation. We established a risk‐score formula as follows: 0.1357 × CENPA + 0.0259 × KIAA1524 + 0.0069 × G6PD + 1.1930 × RPS6KA6 − 0.0026 × PKM. Based on the median risk score, we divided patients into low and high risk‐score subgroups in the TCGA HCC dataset and ICGC‐LIRI‐JP cohort. To have a better prediction of the prognosis in HCC patients by combined utilization of our predicted risk score and clinical indexes, we generated the time‐dependent receiver operating characteristic (ROC) curves by survival ROC R package and integrated univariate, multivariate Cox regression analyses, and nomogram with risk score, *T* stage, *N* stage, *M* stage, TNM stage, age, and gender.^24^

### 3.5 Tumor immune microenvironment assessment

The relative number of 22 immune cells was evaluated by the CIBERSORT algorithm in each case to probe into the correlation of the low risk score group with the high risk score group.^25^ We also used the SSGSEA algorithm to compute the relative expression of CD4 and CD8 cells. The “estimate” R program was used to determine each patient's immunological score, which represented the diversity of immune cell genetic markers.^26^ In addition, the high and low risk score groups shared seven common immune checkpoints, such as mRNA expression, DNA methylation, amplification frequency, and deletion frequency.

### 3.6 Single‐cell data analysis

The analytical pipeline of single‐cell data was carried out as described previously.^27^ Briefly, in order to detect the senescence GSVA score between the HCC and healthy samples in the single cell level, nine HCC samples from GSE 125449 were processed by R package “Seurat.”^28^ The cell filter condition was set to >300 as the screening criterion. After removing mitochondrial genes and extreme values, the expression in the single‐cell level was standardized using “LogNormalize” in the R package Seurat applied to. To further reduce dimensions and annotate cells, R package “Umap” and “SingleR” was used. In addition, to find which cell types the selected 15 TP53‐related DEGs locate in, the heat map was used to display the distribution of expression levels of CENPA, KIAA1524, RPS6KA6, G6PD, CDKN2A, IFNG, PKM, MMP9, SFN, IL1A, and ALOX15B in each subtype of cells; the other four genes were deleted in the previous data processes.

### 3.7 Tumor mutational burden and microsatellite instability calculation

Tumor mutational burden (TMB) and microsatellite instability (MSI) scores in the TCGA HCC database were calculated and acquired in the TIDE platform (http://tide.dfci.harvard.edu/login/), which is a tool for predicting immune checkpoint inhibitor responsiveness.^29^

### 3.8 Patients in TCGA HCC database response to immunotherapy

The response to tumor immune dysfunction and exclusion (TIDE) score, which is used to predict the immune checkpoint inhibitor response and macrophage was calculated and downloaded from the TIDE platform. Then the TIDE score was used to calculate and judge whether each sample from the high‐ and low risk‐score groups is sensitive to PDL1 and CTAL4 treatment in the submap platform, which is an algorithm for making inferences by comparing the similarity of expression profiles.^30^

### 3.9 Clinical samples in the affiliated hospital of Nantong University

Eighty‐four HCC patient samples from individuals who had not undergone preoperative radiotherapy, immunotherapy, or chemotherapy were harvested at Nantong University (NTU) from 2013 to 2022. After collection, samples were stored at −80°C. This study was approved by the Ethics Committee of NTU with every patient having provided written informed consent. Ethics approval is listed in the National Natural Science Foundation of China (No. 81272708).

### 3.10 Western blot analysis

Extraction of protein and Western blot (WB) assays were performed as previously described.^31^ Primary antibodies targeting the following proteins were used in our WB analysis: G6PD (Proteintech, 25413‐1‐AP, 1:1000); PKM (Proteintech, 10078‐2‐AP, 1:1000); CIP2A (KIAA1524, Proteintech, 23199‐1‐AP, 1:1000); CENPA (Abcam, ab45694, 1:1000); RSK4 (RPS6KA6, Abcam, ab76117, 1:1000); CD206 (Proteintech, 60143‐1‐Ig, 1:1000); INOS (Proteintech, 18985‐1‐AP, 1:1000); and GAPDH (Proteintech, 60004‐1‐Ig, 1:10,000).

### 3.11 Multiplex immunofluorescence immunohistochemistry

The detailed procedure was performed in accordance with the manufacturer's instructions.^32^ The multiple immunofluorescence kit was purchased from Record Biology Technology. The channels and concentration for the primary antibodies are listed below. Opal 480 is used for PKM (1:500), Opal 520 is used for RSK4 (1:500), Opal 570 is used for CIP2A (1:200), Opal 620 is used for CD68 (Proteintech, Wuhan, China, 28058‐1‐AP, 1:2000), Opal 690 is used for G6PD (1:500), and Opal 780 is used for CENPA (1:250).

### 3.12 Statistical analysis

All the statistical data analyses were carried out and generated via R software 4.0.3; all the plots were produced from the R package “ggplot2.”^33^ Quantitative values for every experiment were expressed as the mean ± SD. Student's *t*‐test or the Wilcoxon test was used for data evaluation. Statistical significance was exhibited as follows: ns, not significant; *p < 0.05; **p ≤ 0.01; ***p ≤ 0.001.

## 4 RESULTS

### 4.1 Senescence is a character of TP53 mutation in HCC patients

**FIGURE 1:**
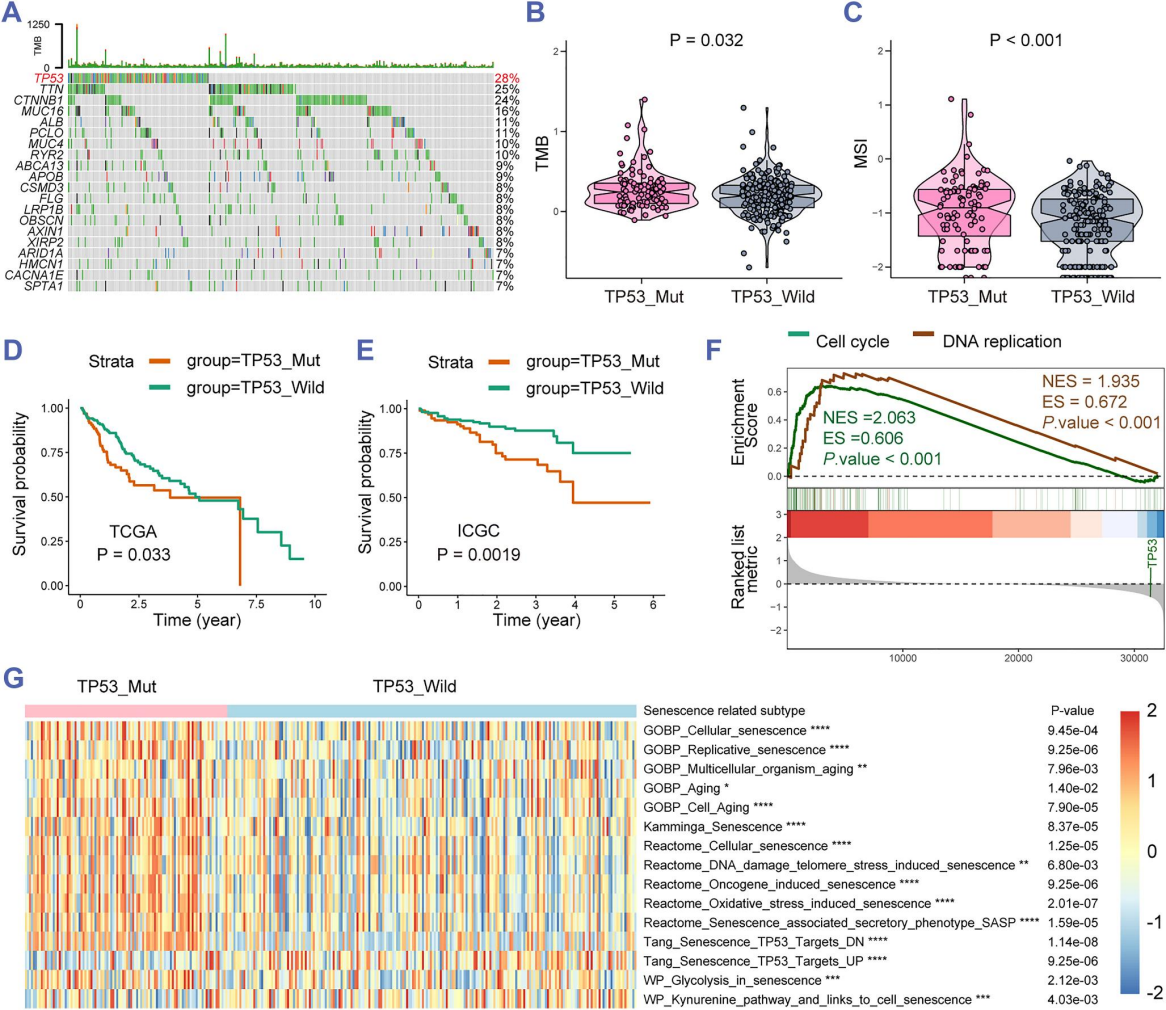
Senescence is a character of TP53 mutation in HCC patients. (A) Mutation states of top 20 genes in the TCGA HCC dataset. (B,C) TMB and MSI state in the TP53 status in the TCGA HCC dataset. (D,E) Kaplan–Meier survival curves based on the prognostic value of the probability of survival with the TP53 status in the TCGA and ICGC dataset. (F) GSEA for TP53 mutation and the wild group in the TCGA HCC dataset. (G) GSVA enrichment displaying the senescence pathways with TP53 mutation and the wild group in the TCGA HCC dataset. GSEA, gene set enrichment analysis; GSVA, gene set variation analysis; HCC, hepatocellular carcinoma; ICGC, International Cancer Genome Consortium; MSI, microsatellite instability; TCGA, The Cancer Genome Atlas; TMB, tumor mutational burden.

**FIGURE 2:**
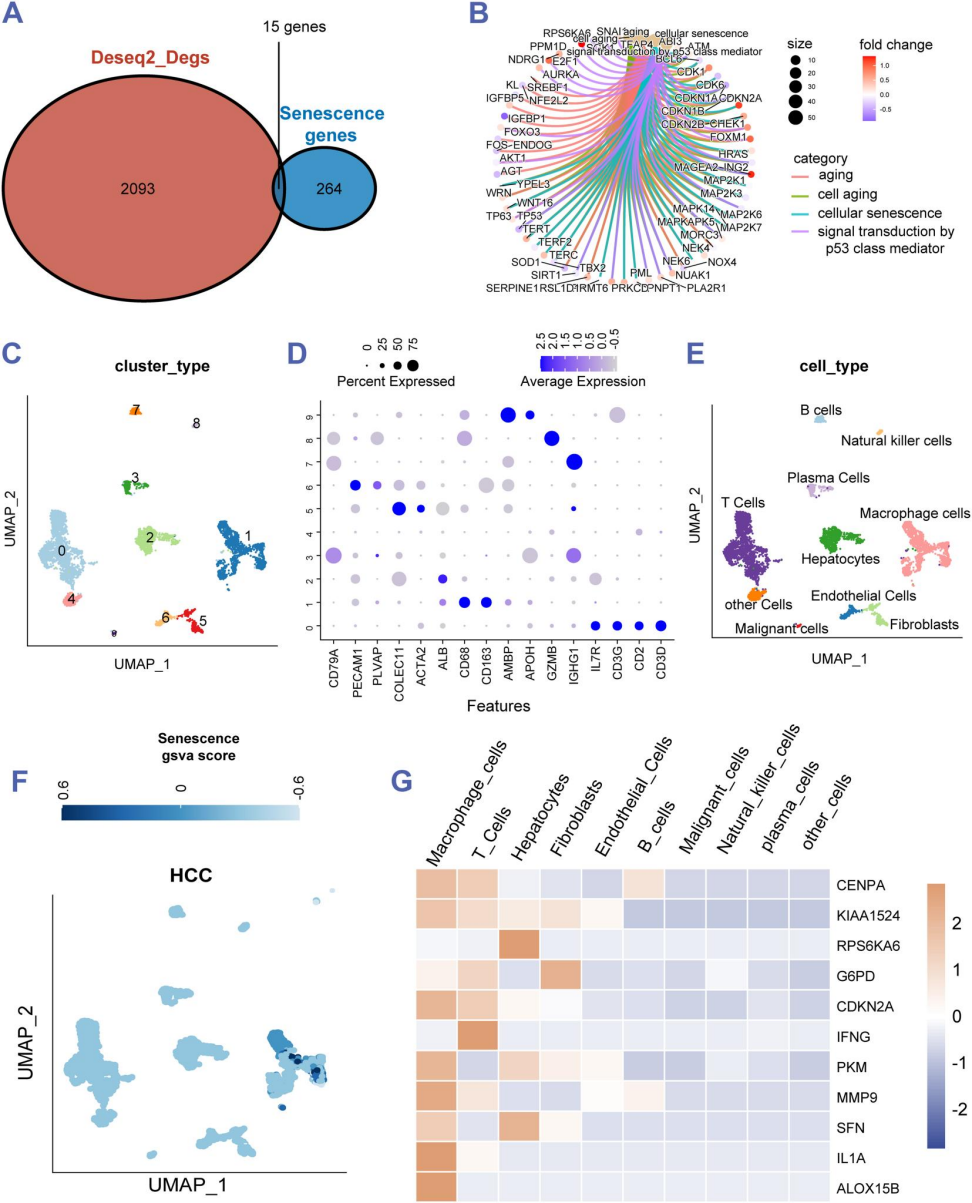
Single‐cell level of senescence related DEGs in HCC. (A) A venn diagram of the DEGs between TP53 mutation and the wild group and the senescence‐related genes. (B) The GO interaction network exhibiting the enrichment of the senescence related DEGS. (C) Single‐cell transcriptomic features for the cell types in GSE125449 is shown in UMAP. (D,E) Marker genes of 10 main cell types identified in GSE125449. (F) UMAP visualization of all cell clusters in GSE125449. (G) A heat map of senescence‐related genes in various cell types from GSE125449. DEG, differentially expressed gene; GO, Gene Ontology; HCC, hepatocellular carcinoma.

Mutations in coding genes are strongly associated with cancer. Hence, we showed the mutational landscape of HCC in TCGA and ICGC databases and found the highest mutation frequency of TP53 in HCC (Figure 2A). As emerging biomarkers, TMB and MSI have attracted more and more attention in predicting the efficacy of tumor immunotherapy. Interestingly, the TMB and MSI scores of TP53 mutation group (*n* = 101) were much higher than the TP53 wild group (*n* = 201) in HCC (Figure 1B,C). To explore the value of the TP53 mutation for prognosis assessment in HCC samples, Kaplan–Meier survival analysis revealed a short overall survival (OS) of HCC patients with TP53 mutation in TCGA and ICGC HCC databases (Figure 1D,E). Furthermore, to elucidate the characteristics of HCC patients in TCGA, GSEA was performed and showed that the cell cycle and DNA replication rank as the top two pathways (Figure 1F). Some recent studies found that telomeres progressively shorten after DNA replication, which results in the replicative senescence and permanent cell cycle arrest.^34^, ^35^ Subsequently, we performed GSVA to explore the correlation of the TP53 mutation with senescence and showed that the senescence‐related pathways are remarkably related to TP53 mutation (Figure 1G). These data indicated that senescence could serve as a prominent hallmark of TP53 mutation in HCC.

### 4.2 Single‐cell level of senescence‐related DEGs in HCC

To explore DEGs that led to the senescence between the TP53 mutation and TP53 wild group, 15 senescence‐related DEGs (MAGEA2, RPS6KA6, IL1A, G6PD, SLC13A3, HEPACAM, KIAA1524, WT1, CENPA, SFN, ALOX15B, IFNG, PKM, CDKN2A and MMP9) were selected by the Venn plot (Figure 2A). Then combined with the selected 15 genes, GO analysis showed these genes may enrich in aging, cell aging, cellular senescence, and signal transduction by p53 class mediator categories (Figure 2B). Furthermore, to find which cell types of the selected 15 related DEGs express in, single‐cell RNA‐seq was generated. After quality control, 10 cell type clusters were identified, including T cells (0, IL7R, CD3G, CD2, and CD3D), macrophage cells (1, CD68, and CD163), hepatocytes (2, and ALB), plasma cells (3, and CD79A), fibroblasts (5, COLEC11, and ACTA2), endothelial cells (6, and PECAM1), B cells (7, and IGHG1), natural killer cells (8, and GZMB), malignant cells (9, AMBP, and APOH), and other unknown cells (Figure 2C–E). Visualized by the umap algorithm, the Reactome senescence GSVA score in macrophage cells was higher than that in the other cell types (Figure 2F). Interestingly, the senescence‐related DEGs were expressed in the macrophage cells, T cells, hepatocytes, and fibroblasts, especially in the macrophages (Figure 2G). Hence, we speculated that TP53 mutation‐related senescence may occur in macrophages of HCC.

### 4.3 Establishment and verification of the TP53 mutation‐related senescence gene prognostic index

**FIGURE 3:**
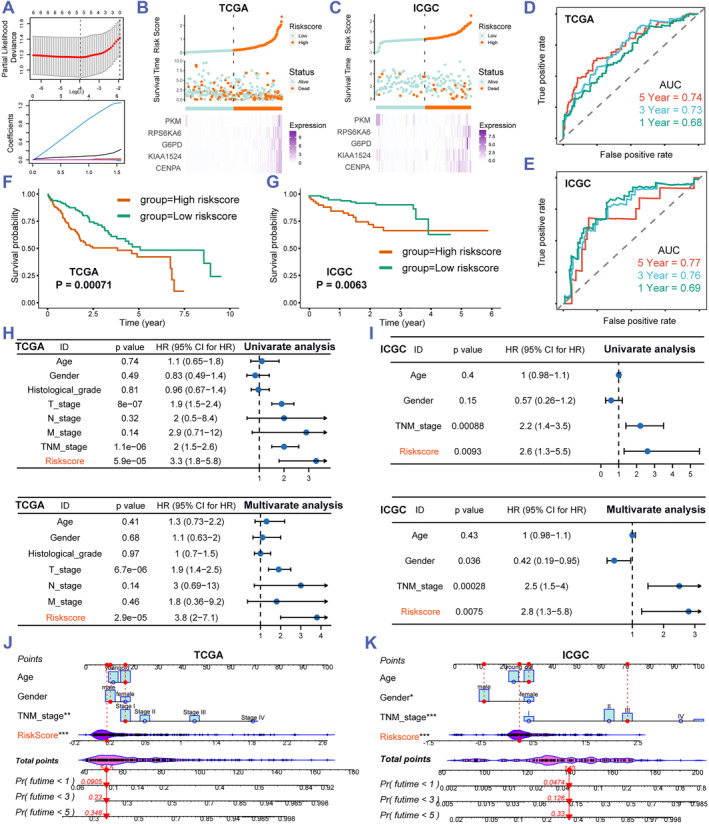
Establishment and verification of the TP53 mutation‐related senescence gene prognostic index. (A) Analysis of cross‐validated tunability parameter selections for LASSO regression models at the best model. (B,C) Distribution of patients based on the risk score and survival status in the TCGA and ICGC dataset. (D,E) Time‐dependent ROC curve analysis for risk score in the TCGA and ICGC database. (F,G) KM curve analysis for risk score in the TCGA and ICGC database. (H,I) Univariate and multivariate Cox regression analysis for risk score and other clinical characters of TCGA and ICGC datasets. (J,K) Nomogram predicts 1‐, 3‐, and 5‐year OS for HCC samples in the TCGA and ICGC datasets. HCC, hepatocellular carcinoma; ICGC, International Cancer Genome Consortium; OS, overall survival; ROC, receiver operating characteristic; TCGA, The Cancer Genome Atlas.

To further study the prognostic value of the selected 15 TP53‐mutation‐related senescence genes, we constructed the prognostic signature in the TCGA HCC database via performing univariate Cox regression analysis and lasso algorithm (Figure 3A). The prognostic model was established with the selected five genes (CENPA, KIAA1524, G6PD, RPS6KA6, and PKM), while the formula is described previously in the methods. Then the TCGA HCC database was utilized as the training set and ICGC‐LIRI‐JP as the validation set. First, the distribution of the five lasso genes' expression, survival status, the risk score, and the KM curve were plotted in the training and validation sets (Figure 3B,C,F,G). These results indicate that the survival rate was lower in HCC patients with high risk score compared with patients with low risk score. Then, the evaluation of the predictive ability in risk score was performed by the time‐dependent ROC curve; the area under curve (AUC) in the training set was 0.68 at 1 year, 0.73 at 3 years, and 0.74 at 5 years, and the AUC in the validation set was 0.69 at 1 year, 0.76 at 3 years, and 0.77 at 5 years (Figure 3D,E). What's more, univariate and multivariate analysis displayed that high risk score is qualified to be an independent factor of short OS in the training set (*p* < 0.001; HR = 3.8; 95% CI: 2–7.1, Figure 3H) and the validation set (*p* = 0.007; HR = 2.8; 95% CI: 1.3–5.8, Figure 3I). Furthermore, we make a prediction of the survival rates in regard to risk score and other clinical characteristics, by formulating a nomogram, indicating that the clinical use of the nomogram in OS prediction was higher than the risk score and the TNM stage (Figure 3J,K). Together, these results suggested that risk score may suffice for a candidate factor in predicting HCC patients' prognosis.

### 4.4 Characterization of senescence microenvironment in low and high risk‐score groups

**FIGURE 4:**
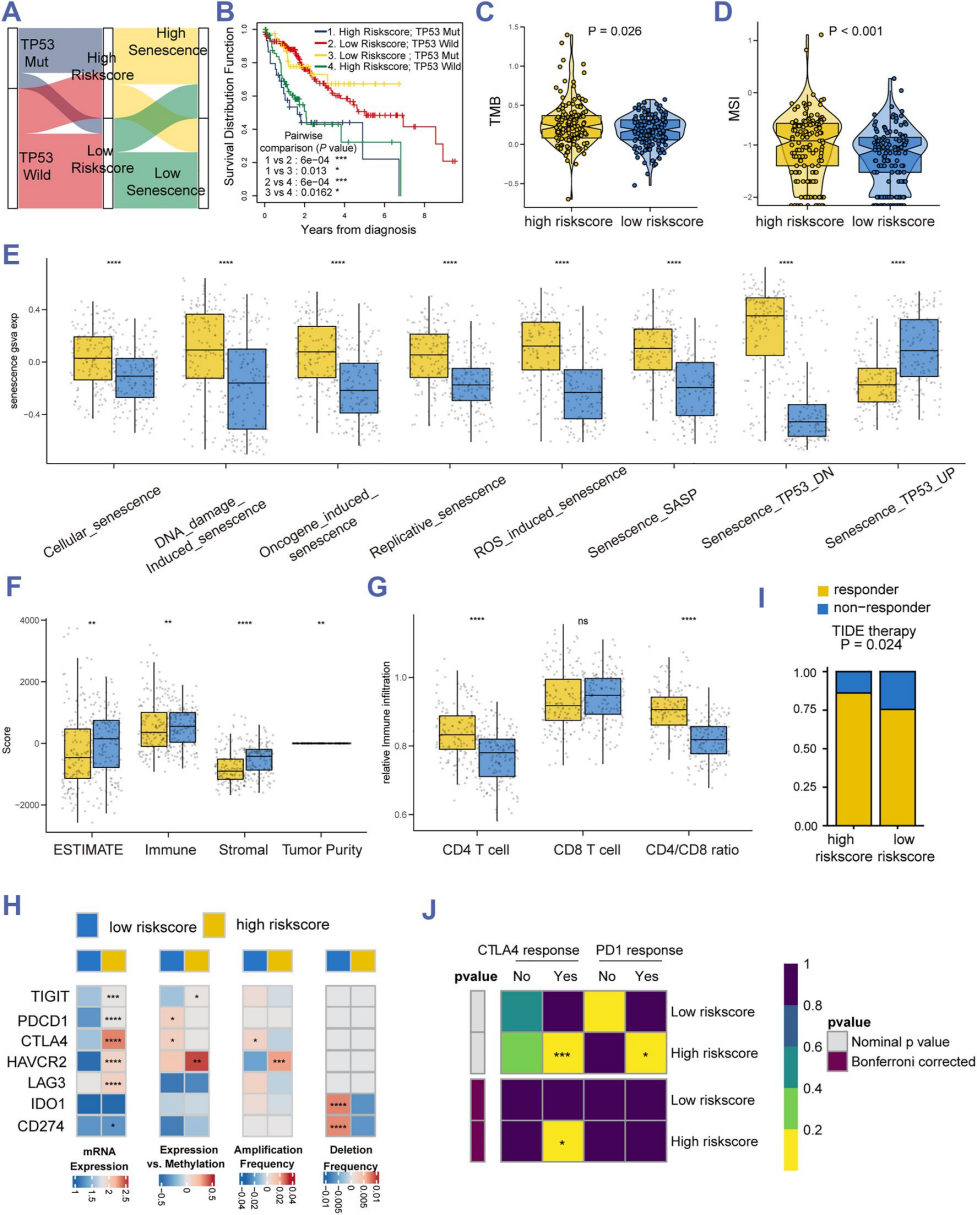
Characterization of senescence microenvironment in low and high risk‐score groups. (A) A sankey diagram revealing the correlation between TP53 mutation, risk score, and senescence score in the TCGA HCC dataset. (B) KM curve analysis for OS in HCC patients who have different TP53 status and risk scores. (C,D) TMB and MSI states in the two risk score groups of TCGA. (E) GSVA analysis for various senescence related pathways in the above two groups. (F) Estimate score for various senescence related pathways in the two groups. (G) Relative active CD4 and CD8 immune infiltration and CD4/CD8 ratio in the two groups. (H) MRNA expression, DNA methylation, amplification frequency, and deletion frequency of seven common immune checkpoints in TCGA. (I,J) Submap analysis manifested a more sensitive response to the anti‐CTLA4 treatment of the high risk score group. GSVA, gene set variation analysis; HCC, hepatocellular carcinoma; MSI, microsatellite instability; OS, overall survival; TCGA, The Cancer Genome Atlas; TMB, tumor mutational burden.

To better illustrate the relationship between the TP53 mutation, senescence, and risk score, a Sankey plot was generated and indicated that patients with TP53 mutation almost have the high risk score and the GSVA senescence score (Figure 4A). For the sake of studying the correlation of TP53 mutation, risk score, and survival, a KM curve was generated and a worse prognosis of patients with high risk score and TP53 mutation than the other subgroups was observed (Figure 4B). Interestingly, senescence is widely recognized as a protective mechanism to fight cancer cells, and senescence in cancer cells may predict better OS.^32^ However, TP53 mutation‐related senescence may result in shorter OS in our study. In addition, we calculated the TMB and MSI scores and discovered that they were much higher in the high risk score group than those in the low risk score group (Figure 4C,D), which indicated that the high risk score group may have the potential for immune evasion. Furthermore, GSVA analysis showed a remarkably activated high risk score in the senescence‐related subtypes (Figure 4E). In addition, several biological processes associated with tumor microenvironment displayed that the high risk score group has a positive correlation with angiogenesis, epithelial mesenchymal translation, glycolysis, and TGFβ, while it has no effect on hypoxia (Figure 1A). To assess the correlation of immune microenvironment with risk score, we used an estimate algorithm and found that the high risk score group has a higher estimate score, an immune score, a stromal score, and tumor purity than those in the low risk score group (Figure 4F). These results demonstrated the changes of different risk score levels in tumor microenvironment, and recent research showed that the senescence of tumor tissues also leads to the senescence of the immune system, especially the conversion of the CD4/CD8 cell ratio.^36^ Interestingly, the SSGSEA algorithm showed that the CD4/CD8 cell ratio in the high risk score group is much higher, indicating a lost response to the cancer cells of the immune system (Figure 4G). Furthermore, the mRNA expression, DNA methylation, amplification frequency, and deletion frequency of seven common immune checkpoints (TIGIT, PDCD1, CTLA4, HAVCR2, LAG3, IDO1, and CD274) were analyzed and a higher mRNA expression of TIGIT, PDCD1, CTLA4, HAVCR2, LAG3, and CD274 was detected in the high risk score group compared with the low risk score group. Interestingly, we found DNA‐methylation of CTLA4 and PDCD1 were higher in the low risk score group, while HAVCR2 was higher in the high risk score group (Figure 4H). Recently, Diane et al. found that patients who have a low CTLA4 methylation level may probably respond to ICB therapy.^37^ To further evaluate whether patients with high risk score can profit from ICB treatment, TIDE and the submap platform were used and showed patients with high risk score may have better therapeutic responses to ICB, especially benefiting from the target of CTLA4 (Figure 4I,J).

### 4.5 The risk score predicts the SASP generated by chemotherapy

**FIGURE 5:**
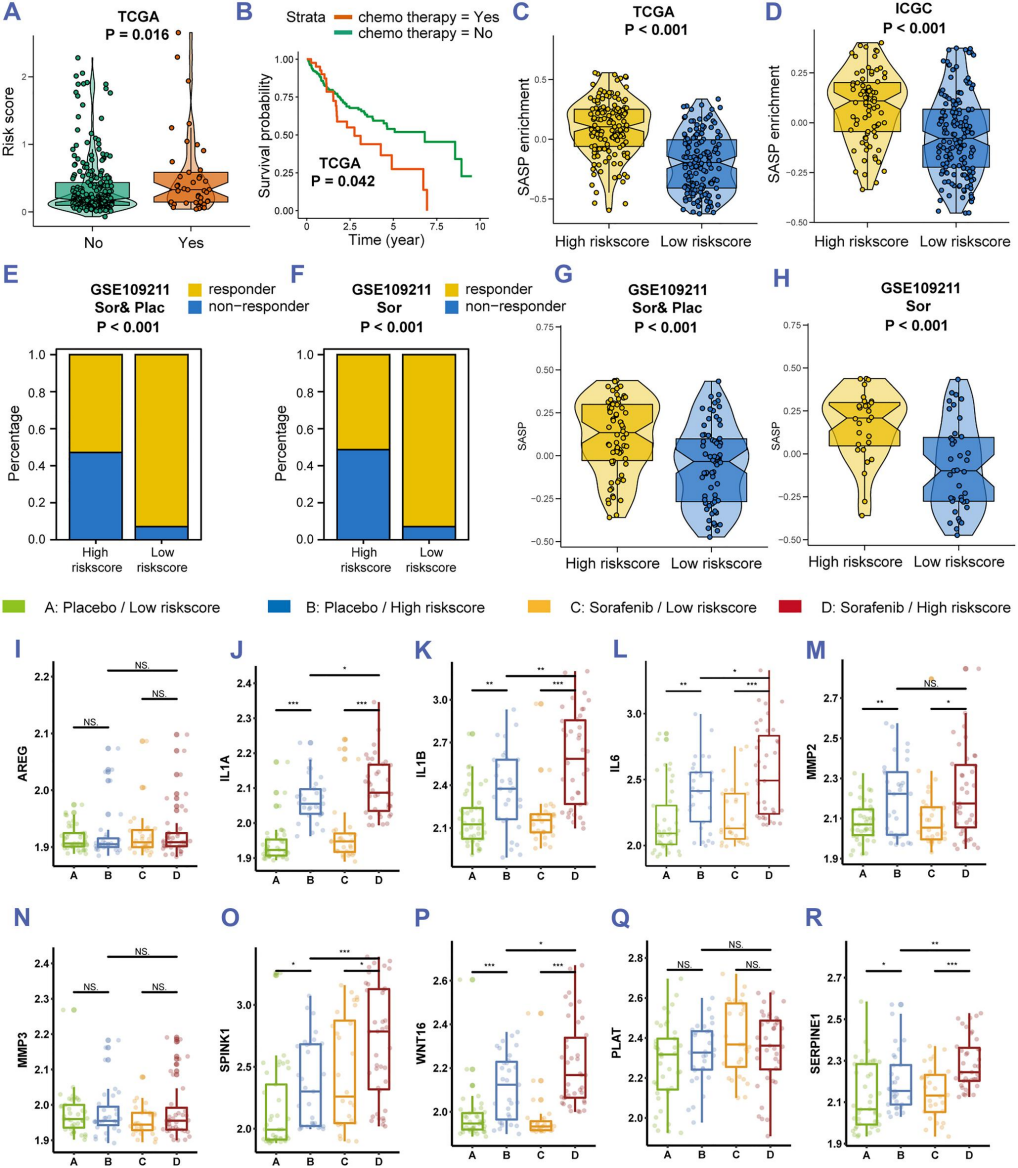
The risk score predicts the SASP generated by chemotherapy. (A) Boxplots of the risk score in the chemotherapy and no chemotherapy groups. (B) The KM curve analysis for OS of HCC patients in the chemotherapy and no chemotherapy groups. (C,D) Boxplots of the SASP GSVA score in two risk score groups of TCGA and ICGC datasets. (E,F) The proportion of patients with drug response in two risk score groups. (G,H) Boxplots of the SASP GSVA score in two risk score groups. (I–R) Boxplots of SASP GSVA score in two risk score groups. GSVA, gene set variation analysis; HCC, hepatocellular carcinoma; ICGC, International Cancer Genome Consortium; SASP, senescence‐associated secretory phenotype; TCGA, The Cancer Genome Atlas.

To evaluate the risk score in chemotherapy, we generated boxplots and found that patients with chemotherapy have a higher risk score than that of patients without chemotherapy (Figure 5A). Interestingly, HCC patients with chemotherapy have worse prognosis than those without chemotherapy, which is opposite to our convention (Figure 5B).In addition, we found that there was no significant difference in survival between chemotherapy and nonchemotherapy patients at the TNM stage I (*p* < 0.05); in TNM Stage II, the long‐term survival rates of chemotherapy patients were lower than that of nonchemotherapy patients (*p* = 0.024). At TNM Stage III, the long‐term survival rates were lower in chemotherapy patients than in nonchemotherapy patients (*p* = 0.013). These results indicated that patients with advanced HCC were not suitable for chemotherapy. Some recent studies showed that HCC cells treated with chemotherapy show more SASP, leading to tumor progression.^18^, ^38^ To further elucidate whether the risk score is correlated with SASP, GSVA was performed, and we found a higher SASP enrichment in the high risk score group (Figure 5C,D). Next, we used GSE109211, a clinical Phase 3 trial, including 67 HCC samples treated with sorafenib and 73, with placebo, and we found that the patients with sorafenib tend to get a high risk score (Figure S2A). Besides, patients who have a low risk score tend to get a drug response, especially in the sorafenib group (Figure 5E,F). As expected, a higher SASP enrichment is observed in the high risk score group, especially in the sorafenib‐treated samples (Figure 5G,H). Moreover, to further evaluate the connection of the risk score with the chemotherapy‐induced SASP genes, the genes (AREG, IL1A, IL1B, IL6, MMP2, MMP3, SPINK1, WNT16, PLAT, and SERPINE1) were obtained.^39^ We generated boxplots and found IL1A, IL1B, IL6, SPINK1, WNT16, and SERPINE1 expressions were higher in the high risk score group compared with those in the low risk score group, and their expression in the sorafenib treated group is higher than that in the placebo‐treated group (Figure 5I–R). Hence, we guessed that HCC cells receiving chemotherapy may release more SASP, which may result in bad prognosis, and the risk‐score could predict SASP progression.

### 4.6 The risk score predicts the M2 polarization of macrophages in HCC patients

**FIGURE 6:**
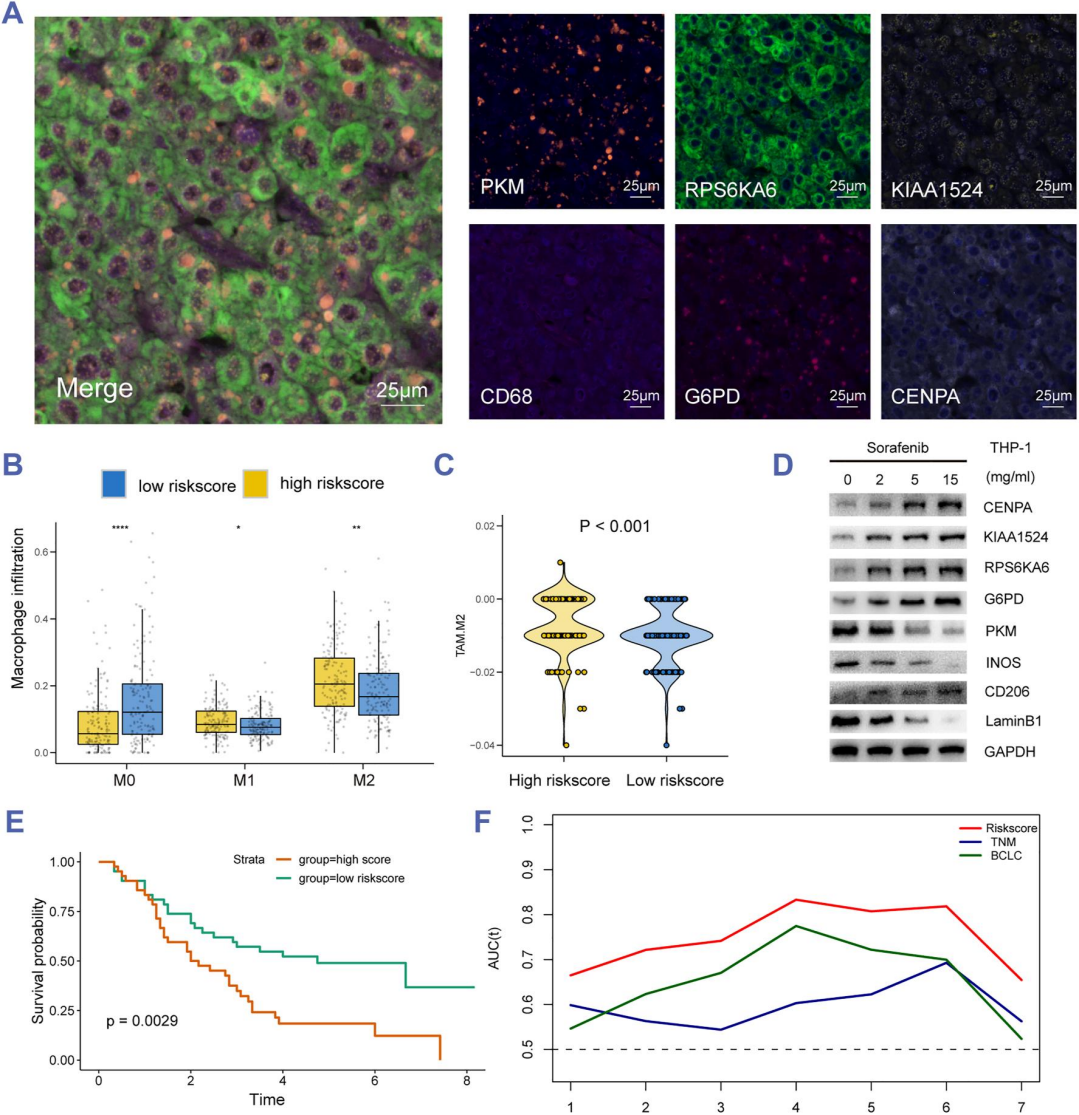
The risk score predicts the M2 polarization in HCC patients. (A) Representative multiplex immunofluorescence immunohistochemistry images displayed the expression patterns of PKM, RPS6KA6, KIAA1524, CD68, G6PD, and CENPA. (B) Relative M0, M1, and M2 macrophages in two risk score groups. (C) Boxplot of M2 macrophage score in two risk score groups. (D) The relative protein levels of risk score genes in sorafenib treated Huh7 cells were detected using western blot. (E) KM curve analysis for OS of HCC patients in two groups from an NTU cohort. (F) A time‐dependent ROC curve for risk score analysis in the NTU cohort compared with the TNM stage and BCLC stage.

From the single‐cell analysis, we speculated that the TP53 mutation‐related senescence genes may be located in the macrophages. To further validate whether the risk‐score genes were located in the macrophages, we used multiplex immunofluorescence immunohistochemistry to identify the protein location of the risk score genes and CD68, a macrophage marker and found they were all located in the cytoplasm of a TP53‐mutated HCC sample (Figure 6A). In addition, the Cibersort algorithm was generated and showed the M2 macrophage infiltration is higher in patients with a high risk score than in patients with a low risk score, while the M0 macrophage infiltration is lower in patients with a high risk score than the patients with a low risk score, which indicates that the risk score was a potential marker in M2 polarization (Figure 6B). Meanwhile, the results from the TIDE platform showed that the high risk score may respond to M2 macrophage (Figure 6C). Moreover, the protein expression of M1 macrophage marker, the risk score, INOS, and M2 macrophage marker, CD206, is analyzed by Western blot. The results demonstrated that upon sorafenib treatment in THP1 cells, the risk score raised higher along with the M2 polarization (Figure 6D). To further study the correlation between clinical characteristics and the risk score, mining with HCC samples from the TCGA database, the ICGC database and the affiliated hospital of the NTU database, we concluded that a high risk score was related to TP53 status and the TNM stage (Table 1). At last, we conducted the KM curve and time‐dependent ROC curve, which revealed the high risk score is an independent element of short prognosis and display more value for OS, compared with the TNM stage and BCLC stage (Figure 6E,F). Collectively, we exhibited that the risk score may become a prospective factor to predict the M2 macrophage polarization, which leads to poor prognosis.

## 5 DISCUSSION

TP53 mutation is the most frequent mutation in HCC and is regarded as the primary driving force for HCC.^9^, ^40^ Similar to previous studies, our results showed that HCC patients with TP53 mutation had poor prognosis than those with TP53 wild status in TCGA and ICGC datasets. Nevertheless, the mechanism of TP53 mutation in accelerating HCC progression is still unclear. Our results showed that TP53 mutation is extremely enriched in senescence‐related pathways. Though cellular senescence is recognized as a protective process to induce cancer cell arrest, emerging evidence has also shown that senescence can also promote the progression of HCC. Mustafa et al. found senescence promotes the increase of stemness in HCC via EpCAM/CD133 pathway, thus producing chemotherapeutic resistance.^38^ At the same time, the senescence of tumor tissues also leads to the senescence of the immune system. This process helps more HCC cells evade immune surveillance, which are supposed to undergo apoptosis.^36^ In addition, large amounts of studies showed the SASPs generated by senescence promote the production of inflammatory factors, which provided the basis for the tumor microenvironment.^41^, ^42^ It is not hard to see that senescence played an indirect role in the process of HCC by shaping the tumor environment. P53 plays an important part in controlling the cell cycle while with the TP53 mutation, HCC cells are prone to senescence. Recently Yukinari et al. pointed out the senescence of TP53 mutated cancer cells release more inflammation cytokines, thus generating heterogeneous tumor‐like cell masses.^43^ Hence, identifying the mechanism of TP53 mutation‐related senescence is vital for inhibiting the development of HCC.

Here, five TP53 mutation‐related senescence genes (CENPA, KIAA1524, G6PD, RPS6KA6, and PKM) were selected, and the prognostic model based on these genes showed the risk score was capable of acting as an independent prognostic factor for HCC. We exhibited 5‐year AUC values of 0.74 and 0.77 of the prognostic model in the TCGA and ICGC cohorts, respectively, consistent with the prognostic utility of this model. The Cox regression analysis and nomogram also verified the superiority of the risk score. Interestingly, these genes play important roles in cancers individually. Xu et al. found that the upregulation of CENPA is positively correlated with poor survival and progression of gastric cancer.^44^ For instance, Pietri et al. found KIAA1524/CIP2A can promote cancer growth by coordinating the activities of MTORC1 and MYC.^45^ Yang et al. found through transcriptional activation of RPS6KA6, FOXP2 could regulate thyroid cancer cell proliferation and apoptosis.^46^ Various studies showed that G6PD leads to enhanced cell proliferation in many types of cancers.^47^ At the same time, G6PD could produce NAPDH to maintain the oxidative balance of cancer cells in the process of glycolysis.^48^ Several studies showed that PKM plays an important role in regulating glycolysis in cancers.^49^, ^50^ Interestingly, Yang et al. considered PKM as one of the three drug targets for HCC patients with TP53 mutation.^51^ The evidence hinted that the five genes are crucial to the progression of HCC. In addition, our data manifested a positive correlation between the high risk score and TMB, MSI, various senescence, tumor environmental related pathways, and stromal score, so we speculated that the risk score could exhibit the formation of tumor environment and contribute to the prediction of HCC patients prognosis.

For HCC patients, surgical resection is the main traditional treatment. However, recurrence and metastasis are the major conundra after treatment, even in patients with early stage HCC.^31^ Fortunately, immunotherapy targeting the immune checkpoints has achieved obvious effects in the treatment of various solid tumors, and some experiments have shown that it has a good clinical effect in the treatment of HCC.^52^ Compared with traditional comprehensive treatment, CTLA4 inhibitors and PD‐1/PDL‐1 inhibitors are more conducive to prolong the survival of advanced HCC patients.^53^ But there are still too many HCC patients who are not candidates for immunotherapy, so it is critical to select the appropriate patients. We investigated the link between the risk score and immune checkpoint genes; results showed that mRNA expression of TIGIT, PDCD1, CTLA4, HAVCR2, LAG3, and CD274 in the high risk score group were higher than those in the low risk score group. Elevated DNA‐methylation levels of CTLA4 and PDCD1 were observed in the low risk score group. Moreover, our results showed patients who possess a high risk score are more likely to benefit from ICB therapy, especially benefit from the target of CTLA4. As such, ICB treatment has the potential to improve high risk score patient prognostic outcomes. Interestingly, our results showed the senescence of the immune system in the high risk score group is also higher via reversing the CD4/CD8 cell ratio. We speculated that senescent immune cells could not react to the occurrence of cancer cells, with the senescence in TP53 mutated HCC cells. All the results showed that a risk score is a promising marker to predict whether HCC patients could benefit from ICB therapy.

At present, most of the HCC patients have reached Stage III to IV when diagnosed. For such advanced patients, the guidelines recommend targeted drugs represented by sorafenib for first‐line treatment.^54^ However, sorafenib has not shown superior survival benefits to HCC patients so far.^55^ Our results showed the HCC patients with chemotherapy have worse prognosis than those without chemotherapy. We also found a risk score is higher in the chemotherapy group. So we speculated chemotherapy could evaluate the risk score, which induced the poor prognosis of HCC patients. In addition to promoting stemness and drug resistance, some studies also indicated that wild‐type p53 preferentially induces senescence rather than apoptosis in chemotherapy‐treated cancer cells, leading to persistent SASP release that drives disease relapse and reduced survival.^56^ Generated SASP can accelerate the progression of cancer via reprogramming the primitive and metastatic microenvironments.^39^ Our results showed the SASP GSVA score in the high risk score group of TCGA, ICGC, and GSE109211 datasets was higher, and we found higher expressions of several chemotherapy‐induced SASP genes in the high risk score group than those in the low risk score group, and the sorafenib‐treated group has higher expressions of these genes than the placebo‐treated group. All these results put forward a novel role of a high risk score as a marker of SASP, which reflects the formation of tumor environment.

Recently, it was previously found that senescence occurred not only in the solid tumor tissue cells, but also in immune cells.^36^ Our results showed that TP53 mutation‐related senescence is more likely to occur in macrophages via single‐cell analysis, which attracted our attention. Rohit et al. elucidated that changes in immune cells associated with senescence are mainly reflected in the phenotype of macrophages, such as the release of inflammatory factors.^57^ Our results showed that the risk score genes (CENPA, KIAA1524, G6PD, RPS6KA6, and PKM) are colocalized with CD68 by multiplex immunofluorescence immunohistochemistry. It implied the risk score genes may play vital roles in the senescence of macrophages. In addition, senescence also leads to M2 polarization.^57^ Our results are also verified using Western blot. Interestingly, M2 macrophages are derived from immature mononuclear cells that circulate through the endothelium under the effect of chemokines, including VEGF, CXCL2, CSF2, and so on, which also serve vital roles in the SASP.^58^ It is well implied that a high risk‐score could also reflect the changes of inflammatory factors of macrophages.

To sum up, this is the first integrated research of TP53 mutation senescence in HCC. Our results supported an independent role of the risk score in predicting HCC patients prognosis. In addition, ICB therapy may most probably benefit HCC patients with a high risk score. Impressively, the relevance of the risk score and SASP in chemotherapy‐treated HCC patients was significant, which implied that a high risk score could act as a marker of SASP during the formation of tumor environment. Lastly, we proved that the risk score is remarkably related to macrophages and M2 polarization As some GEO datasets including the detailed prognosis information could not detect the expression of some of the risk score genes, this limited us to enlarge our patient cohorts. Nevertheless, our research showed that the risk score reflects biological and clinical characteristics relevant to the response upon chemotherapy and ICB therapy. We believe our results will pave a way for developing drug targets for patients with HCC.

## AUTHOR CONTRIBUTIONS

The research was planned and designed by Yi‐Tao Ding, Wen‐Xian Guan, Nan Yi and Hao‐Zhen Ren. The research design was done by Yu‐Yan Chen, Zheng‐Yi Zhu and Tao Ma, and the data was examined by Cui‐Hua Lu. The manuscript was written by Jing Chen and Jia‐Wei Jiang, and it was corrected by Lu Zhang. The article was co‐authored by all authors, and the final text was approved by them all.

## CONFLICT OF INTEREST STATEMENT

The authors declare no conflict of interest.

## ETHICS STATEMENT

This study was approved by the Institutional Research Ethics Committees of the Affiliated Hospital of Nantong University. We confirmed that all methods were conducted in accordance with relevant regulations and written informed consent was obtained from patients.

## Supporting information

Supporting Information S1

## Data Availability

The results of this study are supported by the data available at the TCGA (https://portal.gdc.cancer.gov), ICGC (https://dcc.icgc.org/), GEO (https://www.ncbi.nlm.nih.gov).
