# Supplementary material for: TP53 mutation‐related senescence is an indicator of hepatocellular carcinoma patient outcomes from multiomics profiles
Source: Smart Med. 2023 Apr 13;2(2):e20230005. doi: 10.1002/SMMD.20230005 (PMC11235654; doi:10.1002/SMMD.20230005)
Supplement: Supplementary file 1 — Supporting Information S1 [file SMMD-2-e20230005-s001.docx]

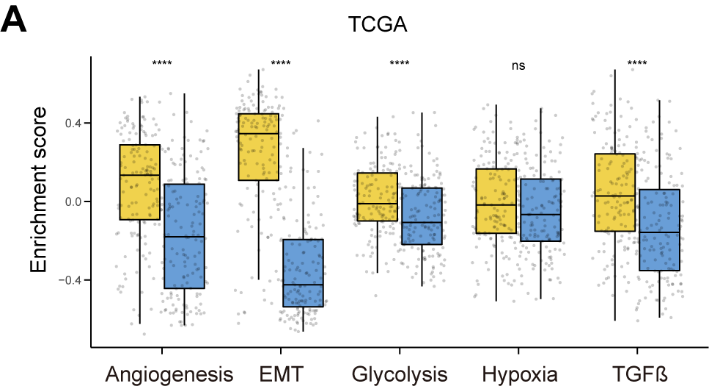


Supplementary Fig1: Characterization of senescence microenvironment in low and high risk-score groups. (A) Boxplot of tumor environment gsva score in low and high risk-score groups.


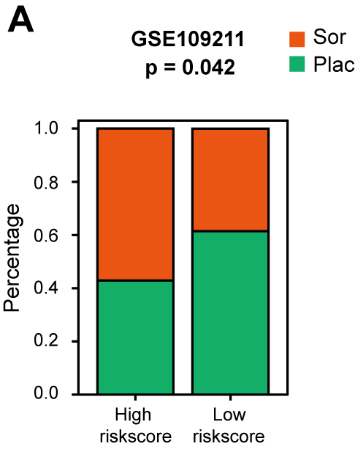


Supplementary Fig2: Risk-score predicts the SASP generated by chemotherapy (A) The proportion of patients with treated drugs in low and high risk-score groups.

**Table1: Clinical characteristics of HCC patients in TCGA, ICGC and NTU databases between the low and high risk-score groups.**

|  | TCGA | | | ICGC | | | NTU | | |
| --- | --- | --- | --- | --- | --- | --- | --- | --- | --- |
| Character | Low | High | *P*.value | Low | High | *P*.value | Low | High | *P*.value |
| TP53 |  | | ＜0.001 |  |  | ＜0.001 |  |  |  |
| TP53 wild | 125 | 76 |  | 96 | 56 |  |  |  |  |
| TP53 mutation | 26 | 75 |  | 24 | 64 |  |  |  |  |
| Age (years) |  | | 0.019 |  |  | 0.07 |  |  | 0.009 |
| ≤65 | 79 | 100 |  | 44 | 41 |  | 27 | 38 |  |
| ＞65 | 72 | 51 |  | 76 | 79 |  | 15 | 4 |  |
| Gender |  |  | 0.169 |  |  | 0.374 |  |  | 0.03 |
| Female | 40 | 52 |  | 27 | 34 |  | 13 | 4 |  |
| Male | 111 | 99 |  | 93 | 86 |  | 29 | 38 |  |
| T stage |  |  | 0.045 |  |  |  |  |  |  |
| T1 | 83 | 65 |  |  |  |  |  |  |  |
| T2 | 39 | 42 |  |  |  |  |  |  |  |
| T3 | 21 | 39 |  |  |  |  |  |  |  |
| T4 | 7 | 5 |  |  |  |  |  |  |  |
| N stage |  |  | 0.604 |  |  |  |  |  |  |
| N0 | 96 | 108 |  |  |  |  |  |  |  |
| N1 | 2 | 1 |  |  |  |  |  |  |  |
| M stage |  |  | 0.236 |  |  |  |  |  |  |
| M0 | 106 | 114 |  |  |  |  |  |  |  |
| M1 | 2 | 0 |  |  |  |  |  |  |  |
| TNM stage |  |  | 0.022 |  |  | 0.944 |  |  | 0.02 |
| Ⅰ | 78 | 63 |  | 19 | 17 |  | 18 | 8 |  |
| Ⅱ | 36 | 38 |  | 55 | 54 |  | 11 | 11 |  |
| Ⅲ | 24 | 43 |  | 35 | 39 |  | 6 | 4 |  |
| Ⅳ | 2 | 0 |  | 11 | 10 |  | 7 | 19 |  |
| Histological grade |  |  | 0.01 |  |  |  |  |  |  |
| G1 | 22 | 16 |  |  |  |  |  |  |  |
| G2 | 85 | 66 |  |  |  |  |  |  |  |
| G3 | 37 | 61 |  |  |  |  |  |  |  |
| G4 | 3 | 8 |  |  |  |  |  |  |  |
| BCLC stage |  |  |  |  |  |  |  |  | 0.190 |
| A |  |  |  |  |  |  | 14 | 7 |  |
| B |  |  |  |  |  |  | 12 | 13 |  |
| C |  |  |  |  |  |  | 16 | 22 |  |
